# From Clot to Cure: Navigating Vascular Pitfalls With Endovascular Salvage of a Delayed Renal Artery Pseudoaneurysm Following High-Energy Renal Trauma

**DOI:** 10.7759/cureus.93070

**Published:** 2025-09-23

**Authors:** Sampangi Raju S P, Ruby Kataria, Madhur Uniyal, Neeraj Kumar, Santhosh Balachandra

**Affiliations:** 1 Trauma Surgery and Critical Care, All India Institute of Medical Sciences, Rishikesh, Rishikesh, IND; 2 Trauma and Acute Care Surgery, All India Institute of Medical Sciences, Rishikesh, Rishikesh, IND; 3 Trauma Surgery, All India Institute of Medical Sciences, Rishikesh, Rishikesh, IND

**Keywords:** blunt renal trauma, coil embolization, covered stent graft, endovascular intervention, renal artery pseudoaneurysm

## Abstract

Renal artery pseudoaneurysm (RAP) is a rare but potentially life-threatening vascular complication of high-grade renal trauma, often presenting with delayed or nonspecific symptoms. While non-operative management (NOM) remains the standard for stable renal injuries, RAP demands timely endovascular or surgical intervention to prevent hemorrhage and preserve renal function. We present the case of a 19-year-old male with a Grade III right renal injury following blunt abdominal trauma, initially managed conservatively. Forty days post-injury, he developed anuria, suprapubic pain, and bladder distension despite an indwelling Foley catheter. Imaging revealed a large RAP (5.3 × 3.6 cm) and a significant bladder clot (9 × 6 × 5 cm). Initial coil embolization of the middle polar artery branch was unsuccessful. Temporary relief was achieved with glue embolization, but recurrent hematuria necessitated the placement of a covered stent graft, which effectively excluded the pseudoaneurysm. The patient remained asymptomatic on follow-up. This case underscores the evolving management of RAP and the limitations of conventional embolization techniques. The progressive approach, from coil embolization to glue and ultimately covered stent placement, achieved durable hemostasis while preserving renal parenchyma. Delayed RAP requires a high index of suspicion, prompt imaging, and a multidisciplinary treatment strategy. Nephron-sparing endovascular techniques like covered stent grafting offer an effective solution in cases where primary embolization fails. This report highlights the critical role of minimally invasive vascular interventions in managing complex renal vascular trauma.

## Introduction

Renal trauma accounts for 1-5% of all trauma admissions, most often from blunt abdominal injury [1]. Despite the retroperitoneal protection, kidneys remain the most commonly injured genitourinary organ [2]. The American Association for the Surgery of Trauma (AAST) renal injury scale classifies these injuries, with Grades III-V considered high grade and often associated with vascular complications [3].

Renal artery pseudoaneurysm (RAP) is a rare but potentially life-threatening complication. It results from incomplete disruption of the arterial wall, forming a blood-filled cavity that communicates with the arterial lumen [4]. Contained by adventitia or perivascular tissue, RAPs may remain silent until rupture. Delayed presentations, ranging from days to months, have been reported, typically with hematuria, anemia, abdominal pain, or hemodynamic instability [5].

Management of renal trauma has shifted toward non-operative approaches in stable patients [6]. However, RAP poses a unique challenge due to delayed hemorrhage and potential renal loss. Failure of embolization necessitates individualized, staged salvage techniques.

We report a rare case of delayed RAP in a young male with Grade III renal trauma, initially managed conservatively but later requiring covered stent graft placement after failed coil and glue embolization. Few reports describe such stepwise escalation, underscoring the importance of vigilance and multidisciplinary intervention in complex renal vascular trauma [7].

## Case presentation

**Figure 1:**
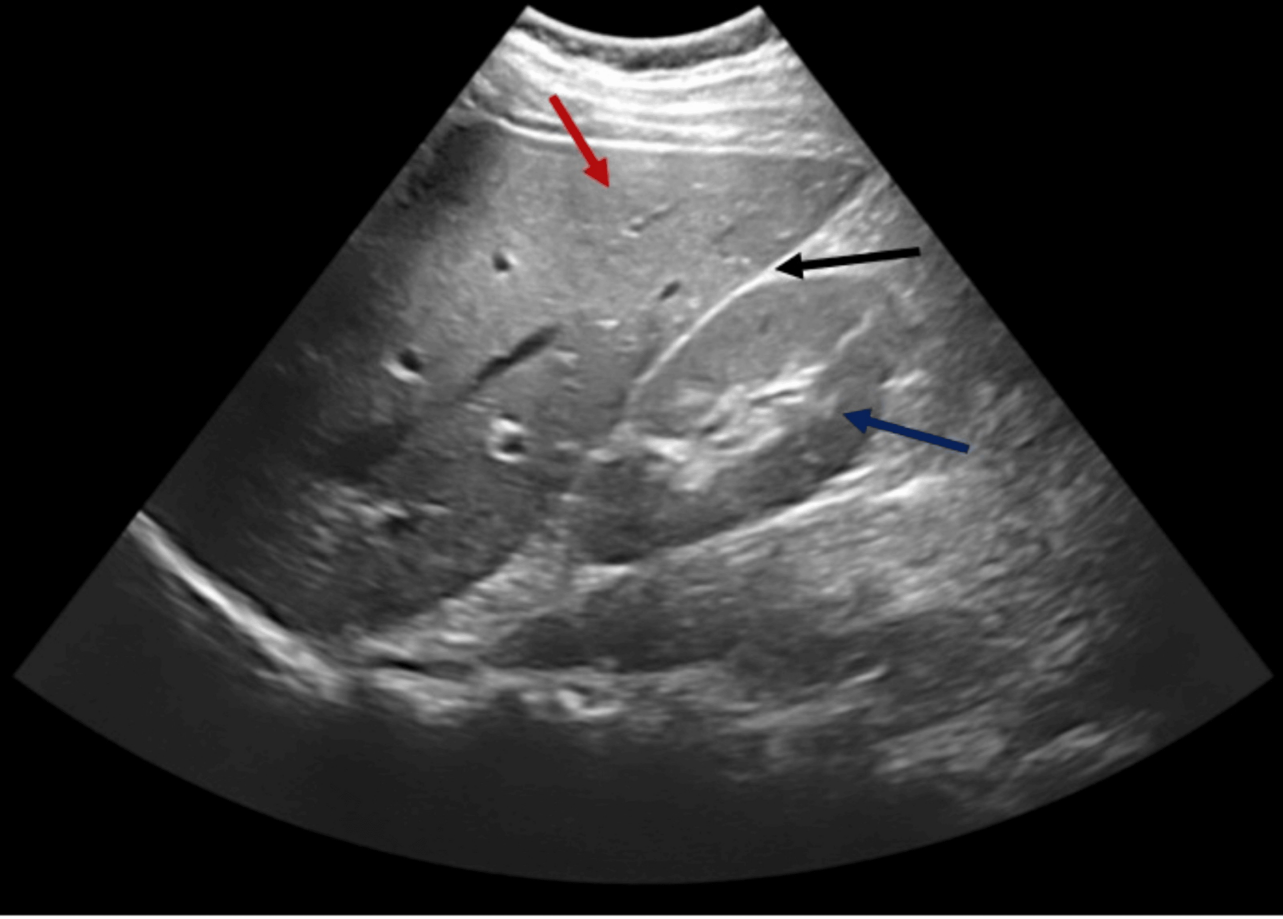
FAST: negative right upper quadrant view. This bedside ultrasound image from a FAST examination demonstrates a negative right upper quadrant view. The red arrow indicates the liver, while the blue arrow points to the right kidney. The black arrow highlights the hepatorenal recess (Morison’s pouch), where no anechoic free fluid is observed. The absence of free fluid in this potential space suggests that there is no intraperitoneal hemorrhage detectable in this view. FAST: Focused Assessment with Sonography for Trauma.

We report a rare case of a delayed RAP presenting 40 days after blunt trauma, leading to life-threatening hematuria and urinary retention. A 19-year-old male sustained a Grade III right renal injury (a >1 cm deep renal laceration without collecting system involvement) from a blunt abdominal trauma six weeks prior. His initial injury was managed conservatively, and he had recovered without surgery. However, approximately 40 days post-trauma, he returned to the trauma emergency department with acute suprapubic pain, visible lower abdominal swelling, and anuria despite having a Foley catheter in place. He reported gross hematuria prior to the anuria. On examination, the patient appeared pale and was in hemorrhagic shock (Class III) with tachycardia and hypotension, indicative of significant internal bleeding. Prompt resuscitation was initiated following Advanced Trauma Life Support (ATLS) protocols; he received aggressive intravenous fluids, blood transfusions, and blood products to stabilize his hemodynamics. Initial bedside ultrasound (FAST: Focused Assessment with Sonography for Trauma) was negative for free fluid in the abdomen, suggesting no intraperitoneal hemorrhage (Figure 1).

**Figure 2:**
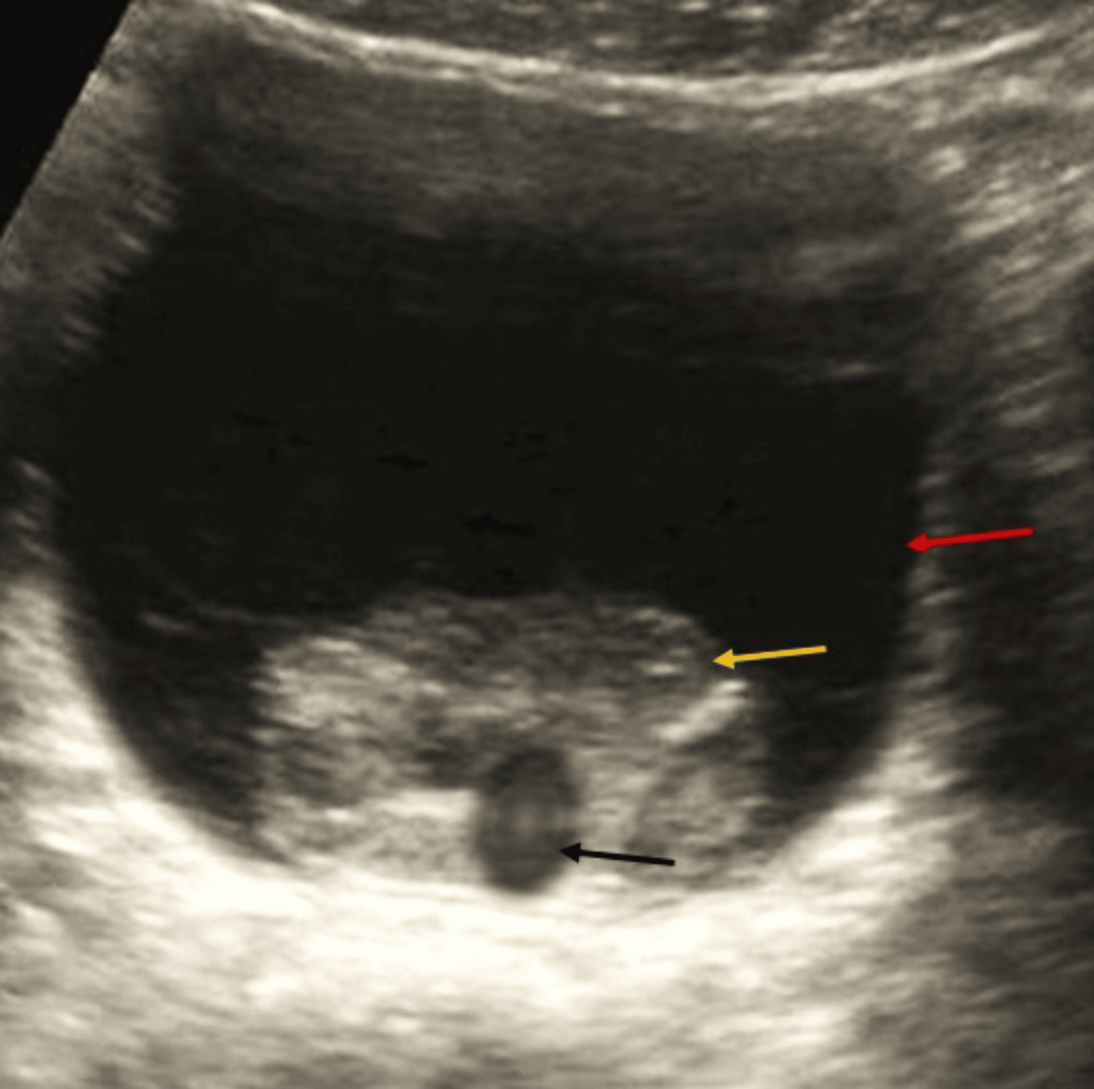
Ultrasound image showing large clot occupying the urinary bladder. This ultrasound image demonstrates a large echogenic clot within the urinary bladder. The red arrow indicates the urinary bladder wall, the yellow arrow points to the echogenic clot occupying the bladder cavity, and the black arrow highlights the Foley catheter bulb in situ.

Notably, the ultrasound revealed echogenic material distending the bladder, consistent with a large clot occupying the urinary bladder (Figure 2).

This explained the patient’s urinary retention despite catheterization. Clot retention in the bladder due to severe hematuria was managed with a three-way Foley catheter for continuous bladder irrigation; however, saline flushing failed to clear the extensive clot. Intravesical thrombolytic therapy was attempted by instilling diluted streptokinase into the bladder. This chemical fibrinolysis helped partially dissolve and evacuate the clot, allowing some urine outflow. Despite partial clearance, the patient’s hematuria persisted, raising concern for ongoing bleeding from an upper urinary tract source.

**Figure 3:**
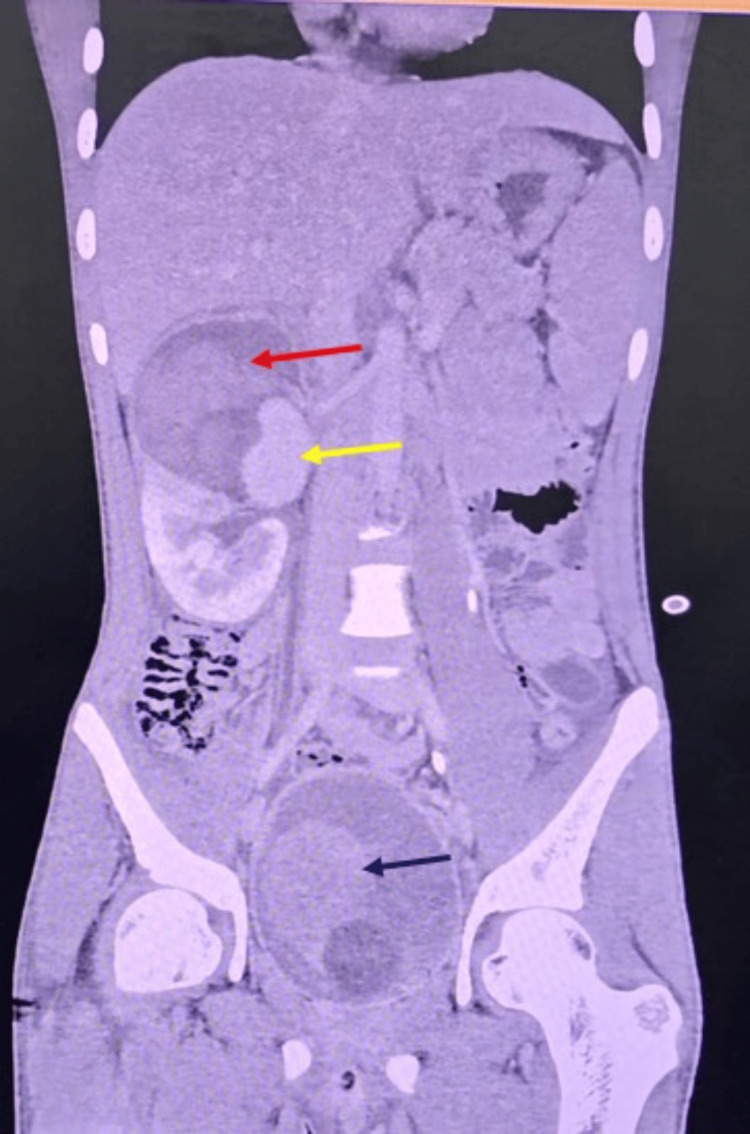
Coronal CECT abdomen showing right renal injury with pseudoaneurysm and bladder hematoma. Coronal CECT image of the abdomen shows a large right perinephric hematoma measuring approximately 9.5 × 8 × 10 cm, with a focal hyperdense area (pseudoaneurysm) indicating active contrast extravasation, consistent with AAST Grade III right renal injury. The red arrow shows the perinephric hematoma; the yellow arrow shows a large pseudoaneurysm arising from the mid-polar region of the right renal artery; and the blue arrow shows a distended urinary bladder with Foley’s catheter bulb in situ, containing ill-defined, non-enhancing hyperdense material in the dependent region, likely representing an intraluminal bladder hematoma. CECT: contrast-enhanced computed tomography; AAST: American Association for the Surgery of Trauma.

A contrast-enhanced CT (CECT) scan of the abdomen and pelvis was obtained to identify the bleeding source. The CT imaging showed that the previous right renal laceration had evolved into a large pseudoaneurysm in the mid-pole branch of the right renal artery. The pseudoaneurysm measured approximately 5.3 × 3.6 cm in size (a substantial vascular cavity) with active contrast filling, confirming it as the likely bleeding source. The surrounding kidney parenchyma remained perfused, corresponding to an AAST Grade III injury without total devascularization. No extravasation of contrast into the abdominal cavity was seen (consistent with the negative FAST). The CT also visualized a large persistent bladder clot (about 9 × 6 × 5 cm) occupying the bladder lumen, corroborating the ultrasound finding of clot retention (Figure 3).

**Figure 4:**
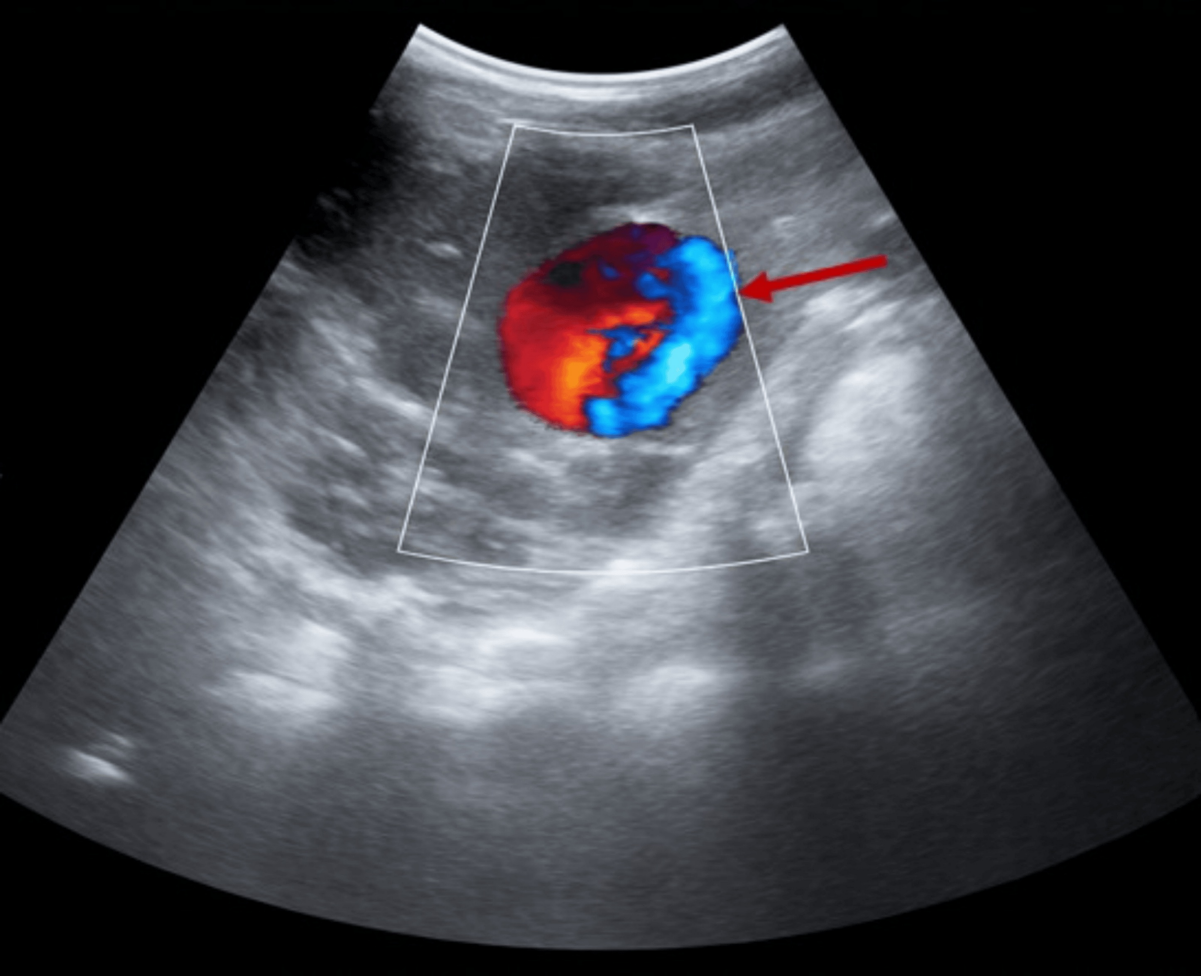
Color Doppler ultrasound of right kidney demonstrating pseudoaneurysm. Color Doppler ultrasound of the right kidney showing yin-yang sign (red arrow) confirming a RAP. RAP: renal artery pseudoaneurysm.

The patient was promptly taken for urgent angiographic management of the pseudoaneurysm in the interventional radiology suite. Prior to invasive intervention, a Doppler ultrasound of the right kidney was performed, which demonstrated the characteristic “yin-yang” sign (a swirling bidirectional blood flow within a vascular cavity), confirming the diagnosis of a pseudoaneurysm on imaging (Figure 4). 

**Figure 5:**
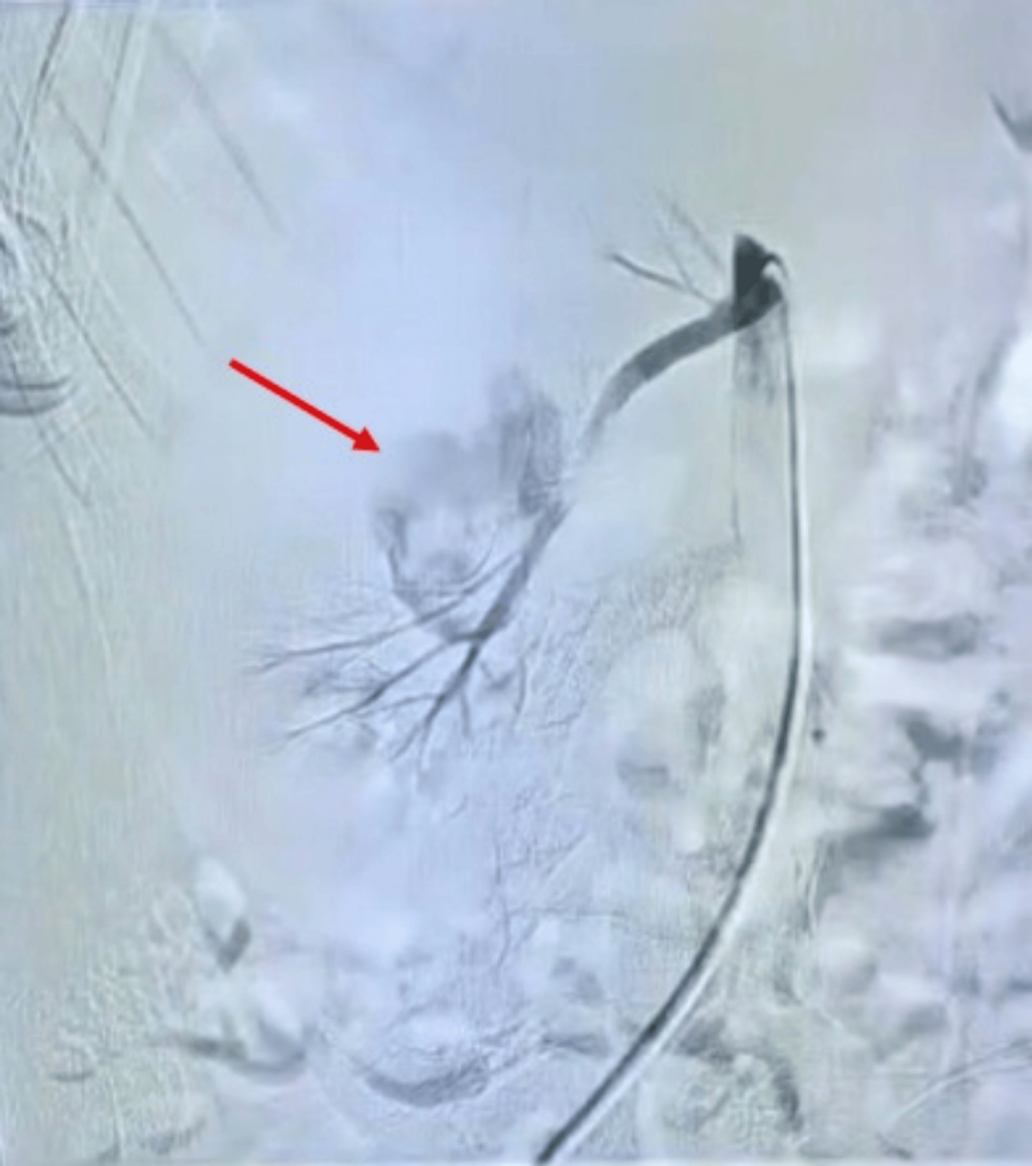
Renal angiography showing a pseudoaneurysm in the right renal artery. This angiographic image reveals a vascular abnormality within the right kidney. The red arrow points to a large pseudoaneurysm arising from the mid-polar branch of the right renal artery. The saccular configuration and localized contrast extravasation are characteristic findings consistent with a pseudoaneurysm.

Renal angiography then pinpointed a large pseudoaneurysm arising from a branch of the right renal artery (mid-polar region) (Figure 5).

**Figure 6:**
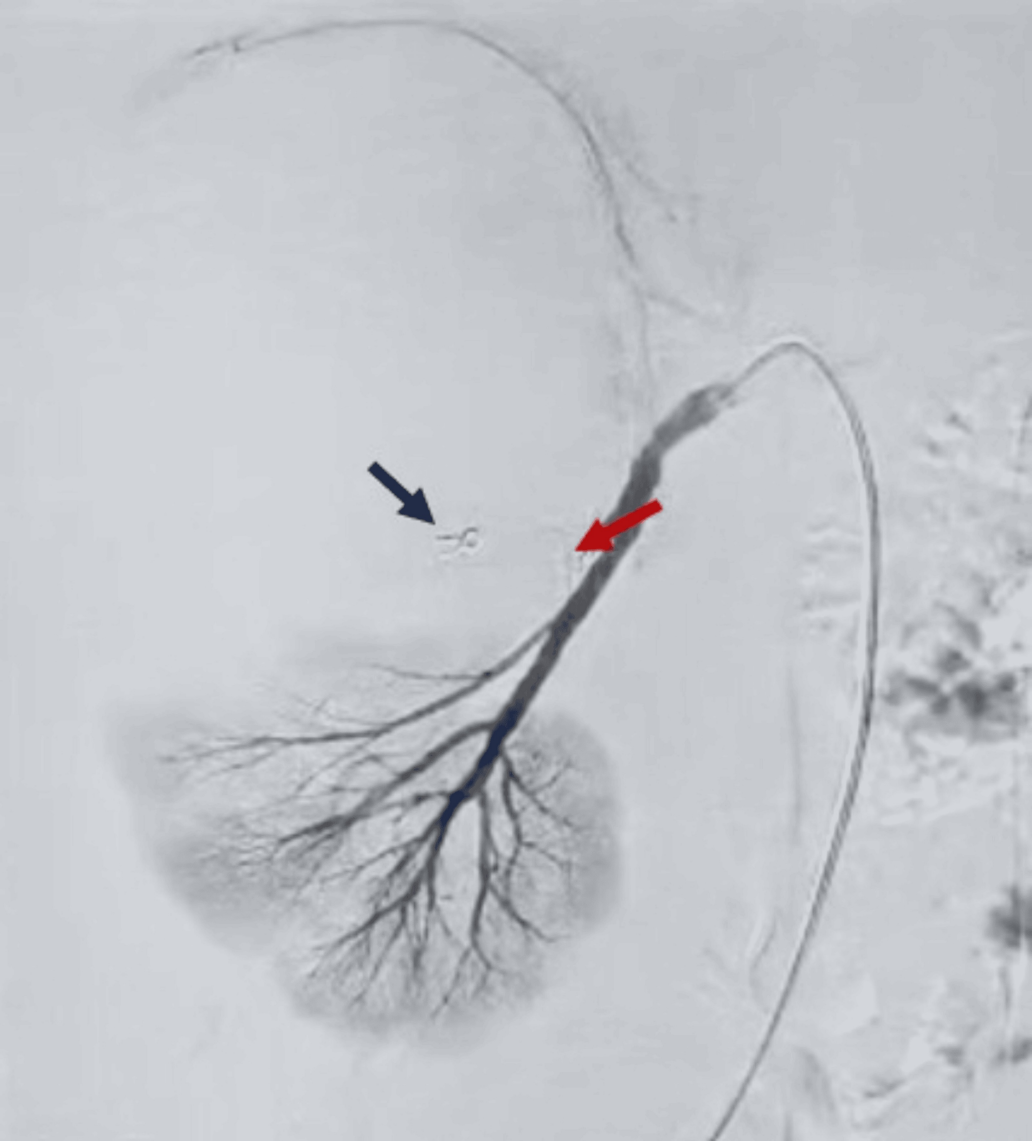
Renal angiography showing selective coil embolization. The renal angiographic image following selective coil embolization demonstrates complete resolution of the previously visualized pseudoaneurysm in the right renal artery. The red arrow shows the disappearance of the pseudoaneurysm, while the blue arrow shows a 3 mm embolization coil deployed at the lesion site.

An attempt was made to treat it with selective coil embolization, which is a typical first-line endovascular therapy for such injuries. Multiple metallic coils were deployed into the feeding artery to induce thrombosis. The initial angiogram post-coiling showed reduced flow into the pseudoaneurysm, and the procedure was considered satisfactory (Figure 6).

**Figure 7:**
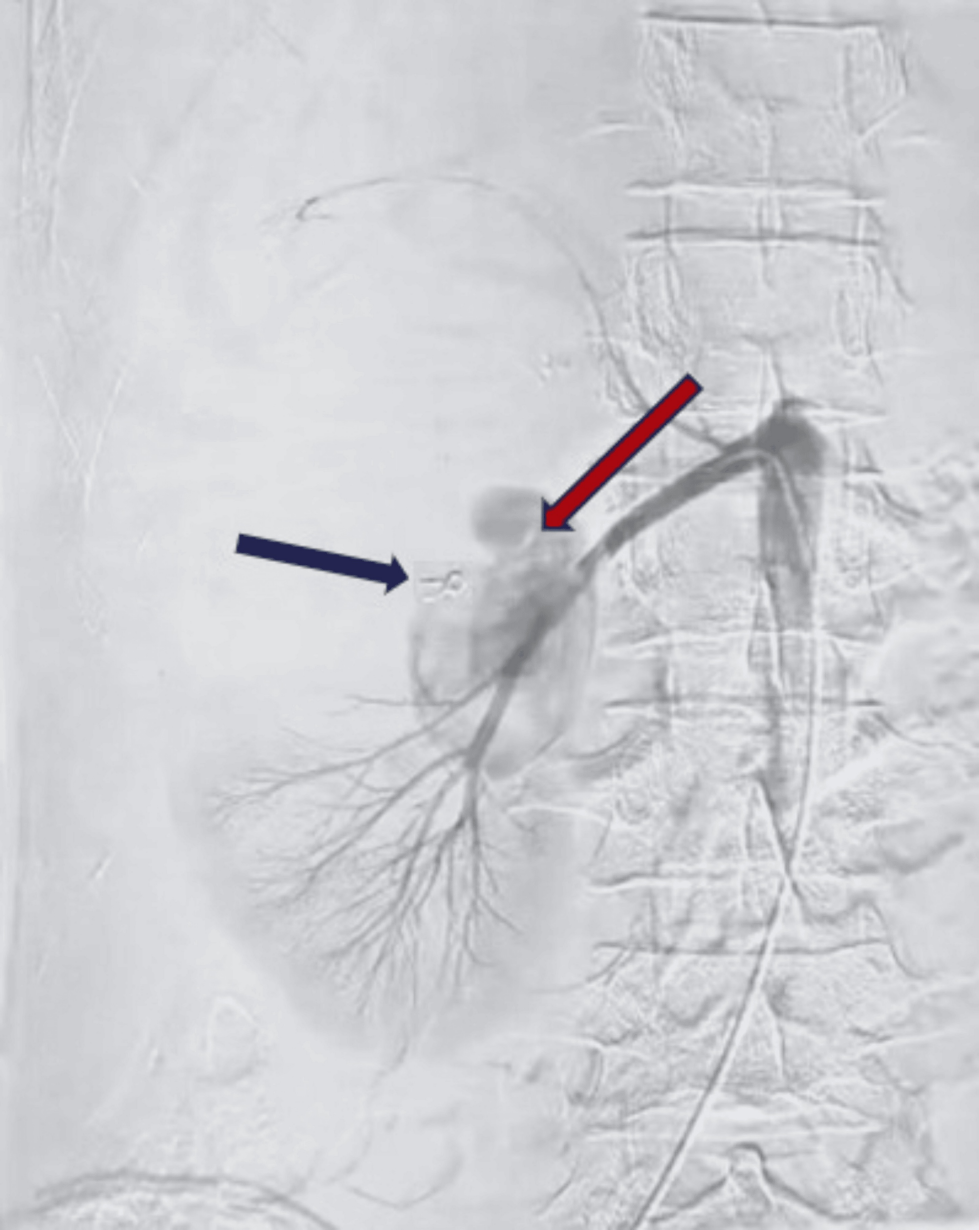
Renal angiography showing reappearance of pseudoaneurysm after failed coil embolization. The renal angiographic image demonstrates the reappearance of a pseudoaneurysm in the right renal artery following prior coil embolization. The red arrow shows the recurrent pseudoaneurysm, while the blue arrow shows the previously placed 3 mm embolization coil.

However, two days later, the patient developed a recurrence of gross hematuria and flank pain, indicating that the coil embolization had failed to completely occlude the lesion. Re-imaging confirmed persistent flow in the pseudoaneurysm sac (Figure 7).

Selective angiographic embolization is generally very effective for post-traumatic renal pseudoaneurysms, so the failure of coil embolization as seen in this case is unusual. Possible causes include high-pressure arterial flow or collateral circulation preventing complete thrombosis of the large pseudoaneurysm. 

Given the rebleed, a second endovascular intervention was undertaken. This time, percutaneous glue embolization was performed. A mixture of cyanoacrylate glue (a liquid embolic agent) was injected through the catheter into the pseudoaneurysm’s feeding artery. The glue embolization successfully sealed off the pseudoaneurysmal cavity on angiography, and the hemorrhage stopped. The patient’s hematuria resolved, and he became hemodynamically stable. With the renal bleeding controlled, the large residual bladder clot was addressed next. The patient underwent cystoscopic clot evacuation under anesthesia: a rigid cystoscope was inserted into the bladder and the organized clot was fragmented and completely washed out. After clearing the bladder, a Foley catheter was left in place for continuous drainage. The patient recovered well from these procedures and was discharged in stable condition with the kidney preserved.

**Figure 8:**
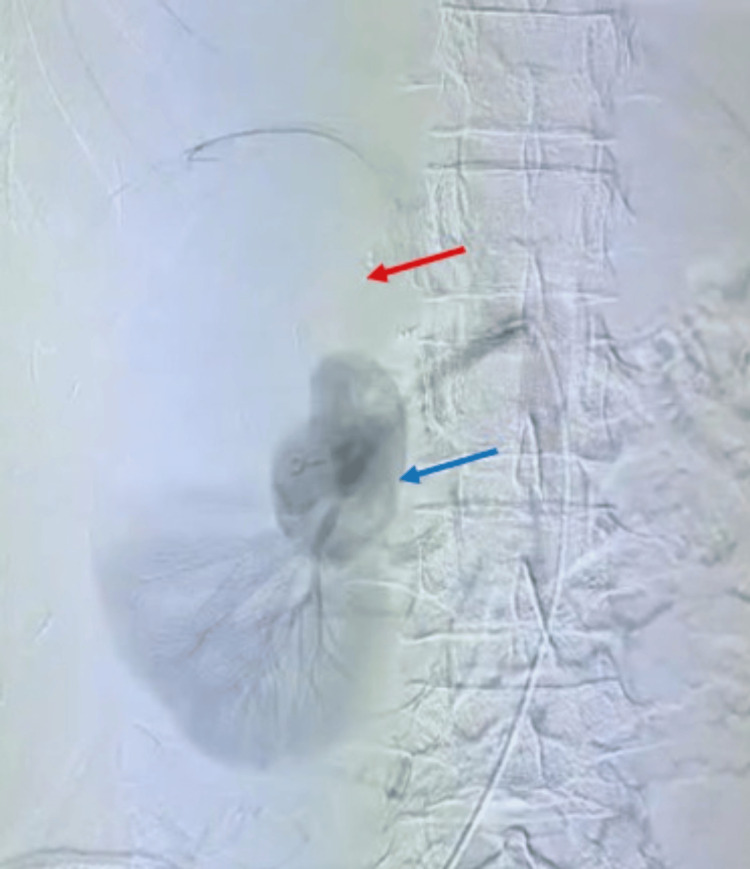
Digital subtraction angiography of the right renal artery pseudoaneurysm (pre-stent graft placement). Digital subtraction angiography of the right kidney, with the blue arrow showing the pseudoaneurysm in the mid-segment of the right renal artery prior to stent graft placement, and the red arrow showing the devascularized portion of the right kidney.

However, four days after discharge, the patient returned with yet another episode of significant hematuria. This recurrence suggested that the glue embolization had failed (either due to incomplete obliteration or reopening of the arterial communication). The case was discussed by a multidisciplinary team, and the decision was made to attempt a definitive endovascular solution rather than proceed to open surgery. The patient was taken for a repeat angiography, which again demonstrated the pseudoaneurysm filling from the mid-pole arterial branch (Figure 8).

**Figure 9:**
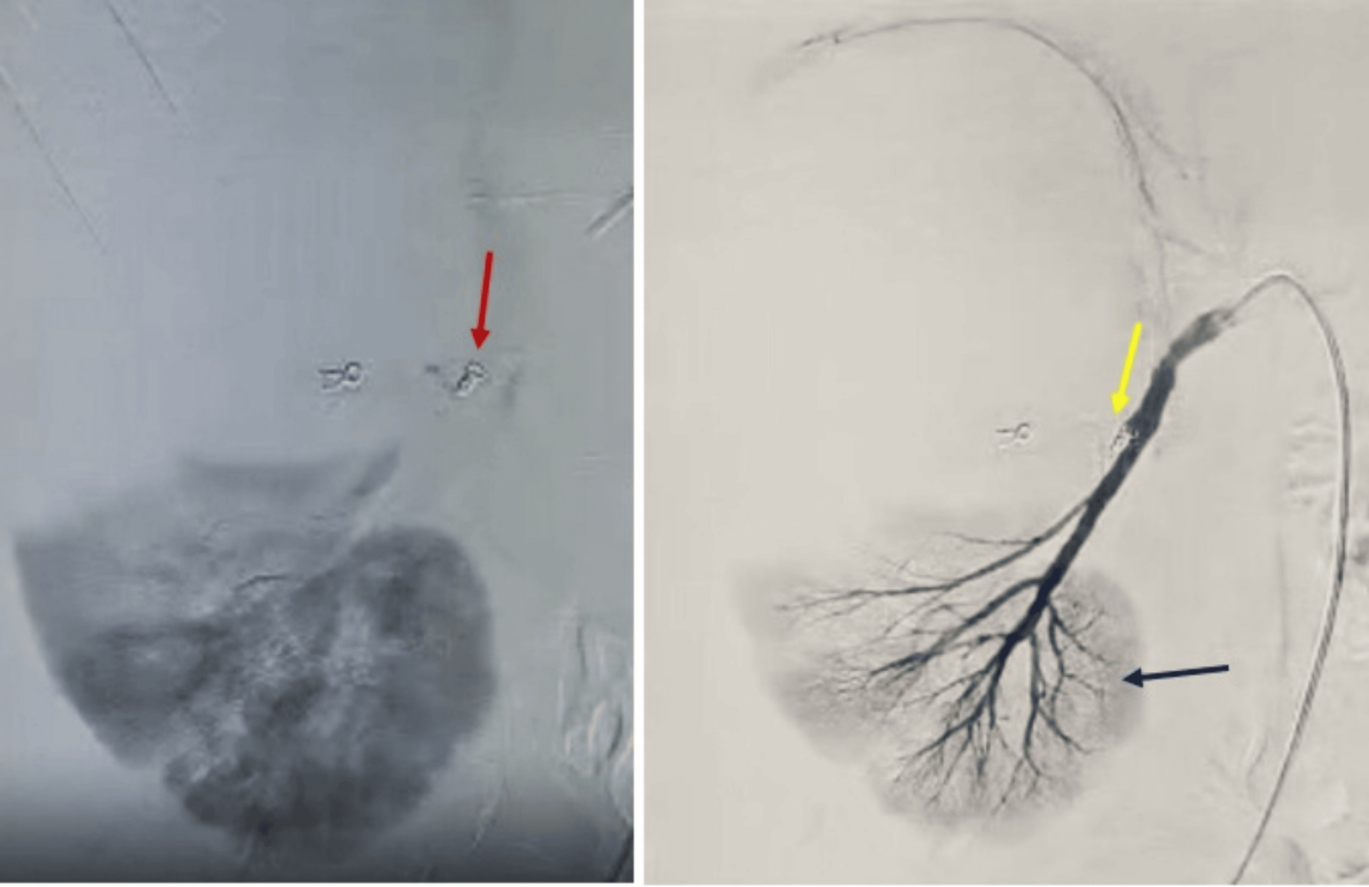
Digital subtraction angiography of the right kidney post-stent graft placement in different phases. Digital subtraction angiography of the right kidney demonstrates post-stent graft placement findings. In the left image, the red arrow shows the covered stent in situ. In the right image, the yellow arrow shows the covered stent effectively excluding the pseudoaneurysm, while the blue arrow shows well-preserved perfusion of the lower pole of the kidney.

This time, an angiographic stent graft placement was performed. A covered stent (balloon-expandable) was deployed across the affected arterial segment, effectively excluding the pseudoaneurysm from the circulation while maintaining blood flow through the parent artery. The post-stent angiogram confirmed that the pseudoaneurysm cavity was no longer opacifying with contrast, and renal perfusion was preserved in the remaining branches of the lower kidney (Figure 9).

After the stent graft, the patient's hematuria stopped completely. He made an uneventful recovery and was discharged home once stable. The patient was followed up for six months after stent placement. Follow-up included clinical evaluations and Doppler ultrasound at two weeks, one month, three months, and six months, which consistently confirmed stent patency without recurrence of the pseudoaneurysm. Additionally, a CECT angiography was performed at three months, which further validated the durable exclusion of the pseudoaneurysm and preserved renal perfusion. Renal function tests (serum creatinine and eGFR) were also monitored at each visit and remained within normal limits throughout follow-up.

## Discussion

RAP is a rare vascular complication most commonly associated with high-grade renal trauma, iatrogenic interventions (e.g., percutaneous nephrolithotomy, renal biopsy), or inflammatory processes [4]. The pathophysiology involves focal disruption of the arterial intima and media, with extravasation of blood that is walled off by adventitia or surrounding tissue, forming a pseudoaneurysm sac. These pseudoaneurysms may be small and asymptomatic or can progress to rupture, leading to life-threatening hemorrhage [5].

In this case, the delayed presentation, 40 days post-trauma, underscores the insidious and unpredictable nature of RAPs. While early imaging might not reveal vascular complications, pseudoaneurysms can evolve over time due to hemodynamic stress or clot dissolution [5]. The presence of a large pseudoaneurysm (5.3 × 3.6 cm) in our patient coinciding with bladder clot retention reflects a late and significant vascular event.

Initial endovascular attempts using coil embolization, the standard modality for RAPs, failed to achieve durable hemostasis [6]. Coil migration, incomplete pseudoaneurysm thrombosis, or persistent collateral perfusion are well-documented causes of failure [8]. The second-tier approach using glue embolization (n-butyl cyanoacrylate) offered temporary resolution, which again failed, demonstrating the limitations of embolization alone in certain anatomical or flow dynamics contexts.

Reported success rates for selective coil embolization in post-traumatic RAPs are high, with most series citing technical success above 90% [8]. However, failures do occur, particularly in the setting of large pseudoaneurysms, high-flow states, or complex branch anatomy, as was evident in our patient. Glue embolization has been used as a salvage strategy, but its durability can be limited, with recurrence reported in up to 10-15% of cases due to incomplete polymerization or recanalization. In this context, covered stent placement offers a valuable nephron-sparing alternative, as it both excludes the pseudoaneurysm and maintains perfusion to the viable renal parenchyma. Our case reinforces that in anatomically suitable lesions, particularly proximal or mid-branch RAPs with recurrent bleeding, earlier consideration of stent grafting may prevent repeated embolization failures, reduce morbidity, and ensure long-term renal preservation.

The eventual use of a covered stent graft was pivotal in sealing the pseudoaneurysm while maintaining renal perfusion [5]. This approach is particularly valuable in middle or proximal branch involvement where parenchymal salvage is desirable. Few older case reports, such as those by Yamaçake et al. and Ekenci et al., have highlighted similar nephron-sparing salvage strategies [5,8]. In recent years, additional reports have echoed these findings. For instance, Kim et al. [9] described a traumatic RAP successfully treated with a two-stage covered stent deployment, achieving complete pseudoaneurysm exclusion by seven months with no stent-related complications. Likewise, Ekenci et al. [8] reported an isolated traumatic RAP managed with an expandable covered stent, noting preservation of renal function without any complications. These contemporary cases reinforce that covered stent-grafts can effectively thrombose renal pseudoaneurysms while sparing renal parenchyma. Moreover, a recent multicenter study by Rossi et al. demonstrated high technical (84-100%) and clinical (84-100%) success rates for covered stenting in visceral artery aneurysms/pseudoaneurysms [10], underscoring the broad efficacy of stent-graft repair in appropriate anatomies. Current guidelines consistently recognize covered stent placement as a valid option for excluding visceral aneurysms/pseudoaneurysms while preserving distal flow in elective or emergency settings.

Long-term data, though limited by the rarity of RAP, are encouraging. Covered stent grafts offer the advantage of maintaining renal blood supply, thereby protecting renal function, as evidenced by multiple case reports of stent-treated RAPs that avoided nephrectomy [5]. Two-year primary patency rates for stented visceral aneurysms vary (approximately 60-100% in the literature) due to differences in vessels and stent types [10]. Importantly, even if a stent occludes over time, it does not usually precipitate renal infarction or loss of function [10]. Recent series have documented covered stent occlusion in 2-16% of treated visceral pseudoaneurysms and even rare cases of extravascular stent migration (2-8%) over long-term follow-up [10]. Notably, these complications tend to be infrequent, and when occlusion occurs, collateral perfusion often prevents clinically evident ischemia. Thus, in survivors of renal trauma or surgery, stent-graft repair not only controls hemorrhage but also maximizes renal unit preservation. Reports of successful stent-graft use in transplant RAPs further highlight the potential for preserving renal grafts that would otherwise be at risk of loss [10]. Overall, judicious use of covered stents, with appropriate sizing and landing zones, has shown durable aneurysm exclusion in most cases, with satisfactory long-term renal outcomes. Close imaging surveillance is still warranted to detect any endoleak, stent stenosis, or late thrombosis that might require reintervention.

The multidisciplinary collaboration in our case, involving trauma surgeons, interventional radiologists, and urologists, enabled a customized treatment approach that preserved renal function without resorting to nephrectomy. Literature supports such tailored escalation: Ahn et al. reported success rates of >90% with angiographic embolization in high-grade renal injuries, but they also emphasized the need for advanced interventional options when embolization fails [11]. This case, in context of the evolving evidence, reaffirms the importance of a high index of suspicion, vigilant long-term follow-up, and readiness for escalation in the face of recurrent or delayed-onset symptoms, even weeks after the initial trauma event [5]. The successful outcome also underlines that timely identification of RAP and the use of novel endovascular techniques like covered stent grafts can salvage renal units that might otherwise be lost, thereby markedly improving patient outcomes.

## Conclusions

RAP is a rare but serious delayed complication of blunt renal trauma. This case highlights the importance of vigilance during follow-up and demonstrates that covered stent grafting provides a durable, nephron-sparing option when embolization fails. Early recognition, timely intervention, and multidisciplinary management remain key to preserving renal function and optimizing outcomes.
